# Versatility and clinical effectiveness of a synthetic sealing hemostatic patch as alternative to parenchyma suturing in laparoscopic partial nephrectomy

**DOI:** 10.1007/s00464-021-08333-0

**Published:** 2021-02-16

**Authors:** Eva Erne, Stephan Kruck, Tilman Todenhoefer, Stefan Aufderklamm, Bastian Amend, Jens Bedke, Arnulf Stenzl, Steffen Rausch

**Affiliations:** 1 Department of Urology, Medical Faculty and University Hospital, Eberhard-Karls-University Tuebingen, Tübingen, Germany; 2 Department of Urology, Siloah am Trudbert Clinic, Pforzheim, Germany; 3 Department of Urology, Eberhard Karls University, Hoppe-Seyler-Strasse 3, 72076 Tübingen, Germany

**Keywords:** Hemopatch, Laparoscopic partial nephrectomy, Veriset, Hemostasis, Complication

## Abstract

**Background:**

Improvements in laparoscopic partial nephrectomy (LPN) in order to minimize perioperative warm ischemia time (WIT), complications, and consequently patient outcome are desirable. Veriset™ is a ready-to-use hemostatic patch of absorbable oxidized cellulose and hydrogel components that has earlier been implemented in vascular and hepatic surgery. We report our experience using this device in LPN.

**Methods:**

Patients with a solitary malignant renal mass suspicious for renal cancer underwent LPN with either the use of Veriset™ hemostatic patch (*n* = 40) or conventional suture technique (*n* = 40). Patient characteristics, operation time and WIT, postoperative course and complications were recorded retrospectively. Tumor complexity was calculated according to the R.E.N.A.L. score. Outcome was determined according to the “trifecta” criteria (negative surgical margin, WIT < 25 min, no complications within 30 days).

**Results:**

No significant differences with regard to clinical parameters and median R.E.N.A.L. score (6) were observed between both groups. Operation time (mean 127.1 min vs. 162. 8 min; *p* = 0.001) and WIT were both lower in the Veriset™ group (14.6 min vs. 20.6 min; *p* = 0.01). No differences in surgical margins (*p* = 0.602) and overall complication rates at 30 (*p* = 0.599) and 90 days (*p* = 0.611) postoperatively were noticed. The surgical outcome according to “trifecta” was achieved in 65% of patients using Veriset™ and in 57.5% of patients by suture closure, respectively.

**Conclusion:**

The hemostatic Veriset™ patch can successfully be implemented in LPN. Handling and application appear favorable, thereby reducing operation time and WIT. The present results suggest that the device may represent an alternative to parenchyma suturing in LPN.

Approximately 99,200 new cases of renal cell carcinoma (RCC) are diagnosed per year and RCC is expected to account for almost 39,100 cancer-related deaths in the European Union in 2018 [1]. The mainstays of curative treatment in localized RCC are partial nephrectomy (PN) and radical nephrectomy. Especially in patients with localized T1 tumors PN is the recommended gold standard treatment [2]. Driven by technical advancements in laparoscopy and progress in the field of robotic-assisted surgery, laparoscopic PN (LPN) is considered a routine procedure in specialized centers. Published data demonstrated comparable oncological outcomes for laparoscopic and open PN [3, 4]. While the benefits of laparoscopic surgery in terms of reduced blood loss and earlier recovery after surgery as compared to open PN have been well documented [5], noteworthy, operation time [3, 4, 6] and warm ischemia time (WIT) are longer during LPN [6]. Since renal function is a main contributor to long-term outcomes after surgery for RCC [7], measures to reduce WIT during LPN are highly desirable. Renal parenchyma closure with laparoscopic sutures may be surgically challenging dependent on tumor localization and complexity and thereby prolong WIT in individual cases. Therefore, the use of easy applicable topical hemostatic substances and wound dressings has been evaluated experimentally and clinically in order to simplify LPN [8, 9]. The hemostatic Veriset™ (Medtronic, Dublin, Ireland) patch is a ready-to-use hemostatic agent made from absorbable oxidized cellulose and hydrogel components. It was initially designed for sutureless tissue closure in vascular and liver surgery [10, 11]. Veriset™ contains no human or animal components and, due to its flexibility, it can be easily inserted into a 10 mm trocar during laparoscopy. It is left in place after hemostasis is achieved and completely absorbed within 4 weeks [12]. Here, we report our initial experience using Veriset™ as a hemostatic layer in LPN and compare surgical results to a contemporary patient cohort undergoing LPN with a conventional laparoscopic suture technique.

## Materials and methods

### Patient characteristics

A total of 85 consecutive patients with suspicious renal mass on computed tomography (CT) or magnetic resonance imaging (MRI) underwent LPN from April 2016 to September 2018 at the Department of Urology, University Hospital Tuebingen. Five patients were excluded: two cases were converted to laparoscopic radical nephrectomy; two cases were converted to an open approach due to massive adhesions; one patient got an additional adrenalectomy. All tumors were single lesions. After tumor excision, in 40 cases a conventional parenchyma suturing was performed (suture group). In the other 40 cases, a Veriset™ patch was applied to the parenchyma defect without additional parenchyma suturing (Veriset™ group).

Patient characteristics and surgical parameters (total operative time, WIT, conversion to open surgery), pathological characteristics and postsurgical outcomes [hemoglobin level, changes in estimated glomerular filtration rate (GFR, CKD-EPI)] were retrospectively recorded from the institutional cancer database. Tumor complexity was evaluated according to the R.E.N.A.L. nephrometry score. Complications within 30 and 90 days after surgery according to the modified Clavien classification [13] were documented and postoperative outcomes according to the “trifecta” criteria (negative resection margin, WIT < 25 min, no complications [14, 15] were compared for both subgroups. In addition, outcome analysis integrating trifecta criteria and renal function preservation as defined by stable GFR of > 90% of baseline value was performed. Written informed consent was obtained by the participants and Institutional Review Board approval for was granted (078/2012B02).

### Description of the surgical technique

**Fig. 1:**
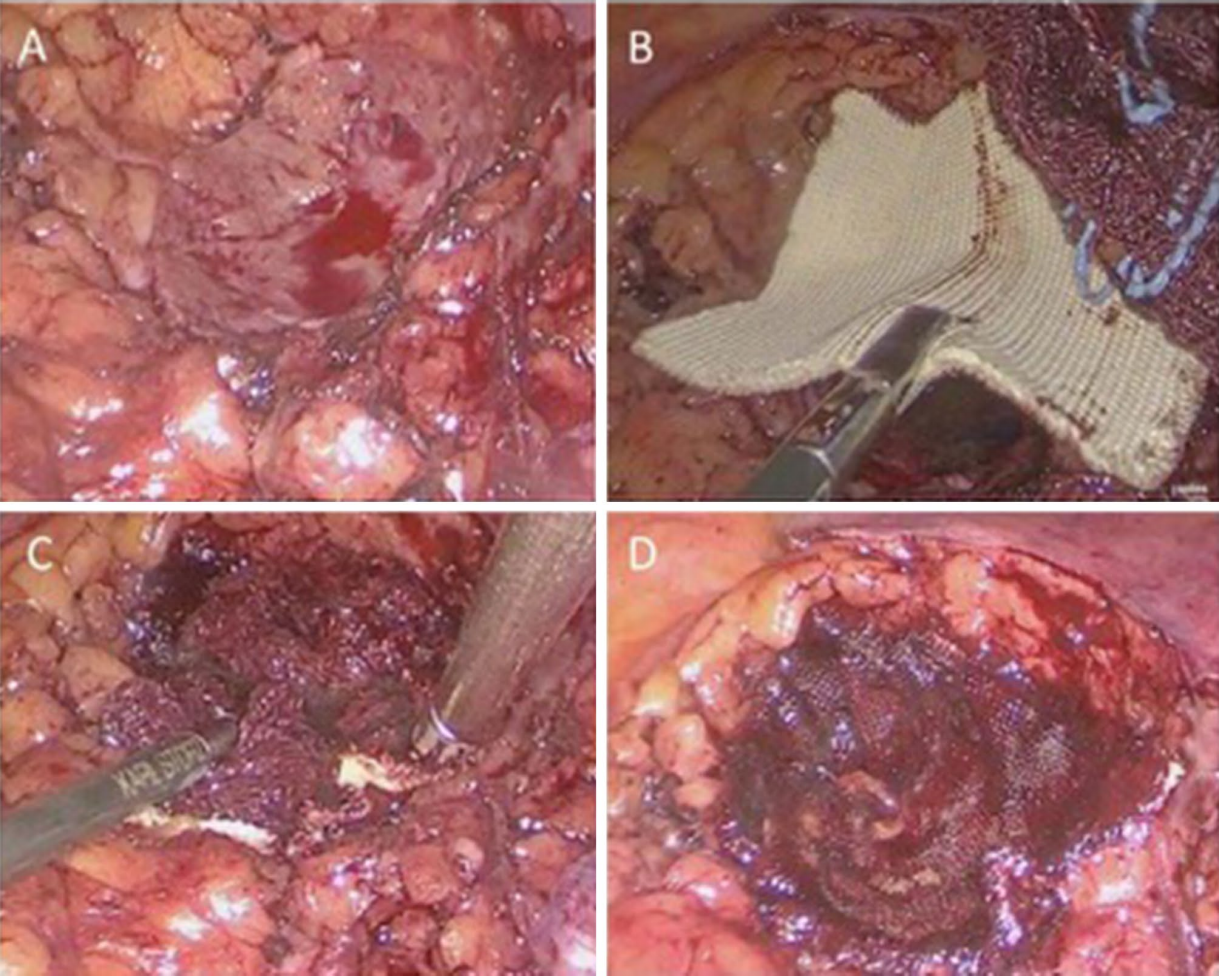
Intraoperative images: **A** resection bed after renal tumor resection, **B** application of hemostatic patch, **C** compression using a moist compress, and **D** final appearance after activation of the patch

LPN was performed by a transperitoneal approach by experienced laparoscopic surgeons. Three ports (one 10 mm, one 5 mm and one 12 mm for the application of the arterial clamp, the Veriset™ device and the retrieval of the specimen) were placed when the lesion was on the left side. An additional 5 mm port was used on the right side to retract the liver. The 10 mm camera trocar was inserted pararectally via a mini-laparotomy and the intraabdominal pressure was adjusted to 12–15 mmHg. The retroperitoneum was exposed by mobilization of the hemicolon and the renal hilus was initially explored and the renal vessels identified and isolated. Hereafter, the kidney was completely mobilized and the tumor was exposed. Warm ischemia was performed by clamping of the renal artery with an endo-bulldog clamp. Afterwards, the tumor was sharply dissected from the surrounding renal parenchyma. If the collecting system was opened, it was closed with a 4-0 polyglactin suture. Selection of parenchyma closure via suture or sealing device was based on preference of the surgeon. For the conventional parenchyma closure, single knot endo-clip polyglactin sutures according to the technique initially described by Lahodny were performed [16]. In the Veriset™ group, no suturing of the parenchyma was performed. The Veriset™ Patch was applied on the parenchyma defect and pressed on for one minute according to the manufacturers’ recommendation. For improved activation and positioning, a moist sterile compress was used to cover the patch and facilitate compression (Fig. 1).

Unclamping was performed after completion of the suture or application of the patch, respectively. In both approaches, the Gerota fascia was reestablished. Finally, the tumor was removed in an endobag.

### Statistical analysis

Statistical analysis was performed using Students’ *t*-test for continuous variables. *χ*^2^ and Mann–Whitney *U* tests were used to compare categorical variables with *p* ≤ 0.05 considered statistically significant. Statistical analyses were performed using commercial software (MedCalc, Version 12.5; Ostend, Belgium).

## Results

The study cohort comprised *n* = 80 patients. The median age of the patients was 62 years, 58.7% of patients (*n* = 47) were men and 41.3% (*n* = 33) women. No significant difference in subgroup composure with regard to clinical parameters was noted. Detailed patient characteristics are shown in Table 1. The median R.E.N.A.L. score was 6 (range 4–10) in both groups. Mean pathologic tumor diameter was 2.7 cm and 3.2 cm in the Veriset™ and suture group, respectively (*p* = 0.113). Overall, 67.5% of renal tumors identified as malignant in the Veriset™ group and 65% in the suture group. A positive surgical margin was detected in one patient each in both cohorts (Table 2).

**Table 1:** Clinical characteristics and postoperative outcome

	Veriset™ group	Suture group	*p*
Number of patients *n* (%)	40 (50)	40 (50)	
Gender; male/female *n* (%)	25 (62.5)/15 (37.5)	22 (55)/18 (45)	0.649
Median age in years (range)	66.5 (37–85)	62.5 (33–82)	0.317
Median/mean tumor diameter in cm (range)	2.3/2.7 (0.7–7.7)	2.7/3.2 (1.0–6.5)	0.113*
Median R.E.N.A.L. score (range)	6 (4–10)	6 (4–10)	0.988
Warm ischemia time in min (mean, SD)	14.6 ± 7.2	20.6 ± 9.6	**0.01**
Operation time in min (mean, SD)	127.1 ± 30.7	162.8 ± 53.6	**0.001**
Median hospitalization in days (range)	5 (4–20)	6 (4–14)	0.408
30-day complication rate *n* (%)	11 (27.5)	8 (20.0)	0.599
90-day complication rate *n* (%)	12 (30.0)	9 (22.5)	0.611
Stable renal function at > 90% GFR *n* (%)	32 (80.0)	27 (67.5)	0.209
Median/mean drop in postoperative Hb (g/dl)	1.1/1.3	1.5/1.4	0.426*
Postoperative outcome according to “trifecta criteria” *n* (%)	26 (65.0)	23 (57.5)	0.579
“trifecta criteria” + stable renal function at > 90% GFR *n* (%)	23 (57.5)	15 (37.5)	**0.025**

Significant values are highlighted in bold

*n* Number, *GFR* glomerular filtration rate, *Hb* hemoglobin, *SD* standard deviation

*p for mean

**Table 2:** Pathological characteristics

	Veriset™ group *n* (%)	Suture group *n* (%)
Clear cell RCC	22 (55.0)	14 (35.0)
Papillary RCC	4 (10.0)	9 (22.5)
Chromophobe RCC	1 (2.5)	3 (7.5)
Oncocytoma	7 (17.5)	5 (12.5)
Angiomyolipoma	3 (7.5)	4 (10.0)
Other benign lesion	3 (7.5)	5 (12.5)
T-stage		
pT1a	23 (57.5)	17 (42.5)
pT1b (*n*)	2 (5.0)	7 (17.5)
pT2a (*n*)	2 (5.0)	0 (0.0)
pT2b (*n*)	0 (0.0)	0 (0.0)
pT3a (*n*)	0 (0.0)	2 (5.0)
Positive surgical margin	1 (2.5)	1 (2.5)
Grading > G2	1 (2.5)	2 (5.0)
NA	13 (32.5)	14 (35.0)

*n* Number, *RCC* renal cell carcinoma, *NA* not available

The mean operative time accounted for 127.1 min in the Veriset™ group and 162.8 min in the suture group (*p* = 0.001), respectively. In both subgroups, three patients were operated under zero ischemia. The WIT was 14.6 min in the Veriset™ group and 20.6 min in the conventional suture group (*p* = 0.01). The collecting system was opened and sutured in 35% (*n* = 14) of patients in the Veriset™ group and in 27.5% (*n* = 11) of patients in the suture group.

Stable GFR > 90% of preoperative level was observed in 80% (*n* = 32) of patients in the Veriset™ group and 67.5% (*n* = 27) in the suture group (*p* = 0.209) and no difference in postoperative drop of hemoglobin level was noted (*p* = 0.426).

Overall complication rates were 27.5% (*n* = 11) in the Veriset™ group and 20% (*n* = 8) in the suture group at 30 days (*p* = 0.599) postoperatively. The 90-day complication rates were 30% (Veriset™; *n* = 12) and 22.5% (suture; *n* = 9), respectively (*p* = 0.611). Further sub-analysis after 30 days revealed two grade III (pseudoaneurysm: *n* = 1; secondary hemorrhage: *n* = 1) and one grade IV (myocardial infarction: *n* = 1) complication in the Veriset™ group and two grade III (urinary extravasation: *n* = 2) and one grade IV (myocardial infarction: *n* = 1) complication in the suture cohort. At 90-day follow-up, in both groups one additional grade III complication was recorded (Table 3). The outcome according to the “trifecta” criteria was observed in 65% (*n* = 26) of patients in the Veriset™ group and 57.5% of patients (*n* = 23) in the suture group (*p* = 0.494). Adding renal function preservation as defined by stable GFR to “trifecta”, the rate of optimal surgical outcome was 57.5% (Veriset™ group: *n* = 23) and 37.5% (suture group: *n* = 15), respectively (*p* = 0.025).

**Table 3:** Complications according to Clavien–Dindo classification

Complications				
	Veriset™ group *n* (%)		Suture group *n* (%)	
	30 days	90 days	30 days	90 days
Grade I	2 (5.0)	2 (5.0)	4 (10.0)	4 (10.0)
Grade II	6 (15.0)	6 (15.0)	1 (2.5)	1 (2.5)
Grade III	2 (5.0)	3 (7.0)	2 (5.0)	3 (7.5)
Pseudoaneurysm	1 (2.5)	2 (5.0)	0	1 (2.5)
Secondary hemorrhage	1 (2.5)	1 (2.5)	0 (0.0)	0 (0.0)
Urine extravasation	0 (0.0)	0 (0.0)	2 (5.0)	2 (5.0)
Grade IV	1 (2.5)	1 (2.5)	1 (2.5)	1 (2.5)

*n* Number

## Discussion

Sutures for the closing of renal parenchyma defects during laparoscopic and robotic-assisted PN represent a standard technique and various approaches have been proposed [17]. However, adequate parenchymal tissue repair remains a challenging step during LPN, with putative detrimental results like intraoperative and postoperative bleeding, urinoma and infection, and also renal function impairment as a consequence of prolonged ischemia [9]. In order to facilitate and optimize hemostasis and parenchyma repair in LPN, several authors have investigated the role of additional hemostatic agents, like TachoSil (Nycomed UK, Oxford, Buckinghamshire, UK), a hemostatic sponge containing human thrombin, or fibrinogen and fibrin glue [18–20].

Veriset™ is a ready-to-use hemostatic patch designed for endoscopic and open surgery. The patch contains of oxidized cellulose and is impregnated with buffer salts, trilysine and a reactive polyethylene glycol and does not contain human or animal coagulation factors [21]. For its activation, the device needs contact with blood and fluids to form covalent bonds with blood proteins and the underlying tissue. Veriset™ amplifies hemostasis via a dual mode of action. On the one hand, it serves as a tamponade to physically stem blood flow, while one the other hand it concentrates and activates platelets and clotting factors to force coagulation [12]. In this study, we evaluated the use of Veriset™ patch as hemostatic layer in LPN and compared its application with our standard laparoscopic suture technique. Most strikingly, our analysis indicates that the operative time and the WIT time were significantly reduced using Veriset™. Regarding the WIT, we suppose that the handling of the hemostatic patch is faster than the suturing of the parenchyma, even by experienced surgeons. Therefore, we observed a significantly decreased WIT. While the rate of postoperative stable GFR at > 90% was observed to be higher in the Veriset™ subgroup (80% vs 67.5%; resp.), this finding was not statistically significant. Since only an insignificant proportion of patients in both groups experienced ischemia time beyond 25 min, and only short-term effects on postoperative renal function were analyzed in the present study, definitive conclusions on the effects on renal function preservation using Veriset™ cannot be made at this point of time. However, Bahouth et al. suggested that suturing the tumor bed is a time consuming step during LPN [22], while Ebbing et al. showed that the WIT is a significant risk factor for acute kidney injury and suggested that clampless PN or at least the shortest possible WIT would reduce the risk impairment of renal function [23]. Zhang et al. also detected that an acute decline in renal function after PN was associated with prolonged WIT, which appeared to impact subsequent functional recovery [24]. However, other authors reported that limited WIT (i.e., ≤ 25 min) did not bear a higher risk of reduced renal function after PN as compared to a ‘zero ischemia’ technique [25]. Despite this controversy, a short WIT will ultimately lead to a limited overall operation time, as also noted in the present analysis, where the operation time was significantly decreased in the Veriset™ group. We assume that parameters like tumor complexity or diameter might also have an impact on operation time. However, in our collective these factors were not significantly different between the respective subgroups.

Regarding postoperative complications and outcomes, in the present analysis, no differences in overall complication rates and the optimal outcome according to “trifecta” criteria were detected using the Veriset™ device. However, when renal function preservation was combined with “trifecta” outcomes, a significant benefit for the Veriset™ technique could be detected. Noteworthy, in both patient subgroups three grade III complications were observed. In the Veriset™ group, two patients developed pseudoaneurysm and one patient had secondary hemorrhage. With regard to postoperative risk of bleeding, it should be reflected that Veriset™ serves as a mechanical hemostat by concentrating and activating platelets and clotting factors for hemostasis. The patch is not biologically active itself and is considered as passive hemostat. In conclusion, for adequate function of the patch, the patients’ own intact coagulation system is a prerequisite [26]. Noteworthy, the patient suffering from secondary bleeding in our analysis was retrospectively found to have a coagulation disorder. While there are no general application restrictions in dependence of routine preoperative patient work-up by bleeding anamnesis, blood count, coagulation and liver enzymes, application of the patch in patients with known tendency to hemorrhage or coagulation disorders should therefore presumably be omitted.

The fact that postoperative pseudoaneurysms were noted in the Veriset™ patient subgroup is another finding that should be closely monitored during the further evaluation of the device. Interestingly, Shigeta et al. proposed that the combined application of a TachoSil™ patch and renorrhaphy led to a reduced incidence of pseudoaneurysms as compared to parenchyma sutures only [19]. Other hemostatic sealing devices, like fibrin glue, were earlier reported to lead to secondary ureteral obstruction presumably due to associated inflammation or adhesion in the peri-ureteral area [18].

In general, application of the patch during laparoscopy was favorable, and no damage of the patch itself or loss of the functional layer after passage through the port was noted. For follow-up studies, Veriset™ owns favorable characteristics, since due to its complete absorption, it should not mimic tumor recurrence in imaging studies.

Finally, there are several limitations looking at the present analysis. First, the design of the study being retrospective and non-randomization of comparison subgroups are limitations that must be acknowledged. Inherent bias due to the fact of surgeons’ preference for the selection of type of parenchyma sealing cannot be excluded, despite no differences in tumor characteristics in the comparative groups. Moreover, the number of cases is overall still limited. Further analysis in randomized trials for LPN appears however warranted, given the favorable results from the present exploratory analysis. Overall, in both groups the mean tumor diameter was small, which should be reflected with regard to the application in larger RCC.

Moreover, only patients undergoing conventional laparoscopy were included. Given the growing caseload of robotic-assisted PN [5], it appears worthwhile to apply the patch in analogy to the present approach using robotic systems as well.

In conclusion, the hemostatic Veriset™ patch can successfully be implemented in LPN. Handling and application appear favorable and the present results indicate time-preserving properties with regard to overall operation time and warm ischemia time. The patch may therefore be an alternative to parenchyma suturing in LPN that warrants further systematic evaluation.
